# Chemical composition, anti-biofilm activity and potential cytotoxic effect on cancer cells of *Rosmarinus officinalis* L. essential oil from Tunisia

**DOI:** 10.1186/s12944-017-0580-9

**Published:** 2017-10-02

**Authors:** Marwa Jardak, Jihene Elloumi-Mseddi, Sami Aifa, Sami Mnif

**Affiliations:** Laboratory of Molecular and Cellular screening Processes, Centre of Biotechnology of Sfax, P. O. Box 1177, 3018 Sfax, Tunisia

**Keywords:** Essential oil, Chemical composition, *Staphylococcus*, Anti-biofilm, Anti-cancer, Fluorescent microscopy

## Abstract

**Background:**

*Rosmarinus officinalis* L. from Tunisia, popularly known as rosemary, is of a considerable importance for its medicinal uses and aromatic value. The aim of this study was to examine the chemical composition of *Rosmarinus officinalis* essential oil (ROEO) and to evaluate its antibiofilm activity on biofilm-forming bacterium and its anticancer activity on cancer cell lines.

**Methods:**

The chemical composition of *Rosmarinus officinalis* essential oil (ROEO) was analyzed by GC-MS and its antibacterial activity was evaluated by micro-dilution method. The antibofilm activity of ROEO was evaluated using the crystal violet test and the cytotoxicity activity was determined by the MTT assay.

**Results:**

In this research, thirty-six compounds were identified in ROEO using GC-MS analyses. The main components were 1,8-cineole (23.56%), camphene (12.78%), camphor (12.55%) and β-pinene (12.3%). The antibacterial activity of ROEO was evaluated by micro-dilution method. The oil exhibited inhibition and bactericidal effect against two strains: *Staphylococcus aureus* ATCC 9144 and *Staphylococcus epidermidis* S61. It was found that the minimum inhibitory concentration (MIC) obtained for *S. aureus* and *S. epidermidis* ranged from 1.25 to 2.5 and from 0.312 to 0.625 μl ml^−1^, respectively and the minimum bactericidal concentration (MBC) were in the order of 5 and 2.5 μl ml^−1^, respectively. Furthermore, this oil showed a *S. epidermidis* biofilm inhibition more than 57% at a concentration of 25 μl ml^−1^. The eradication of 67% of the established biofilm was observed at a concentration of 50 μl ml^−1^ of ROEO, whereas the dose of 25 μl ml^−1^ removed only 38% of preformed biofilm. ROEO strongly inhibited the proliferation of Hela and MCF-7 cells with IC_50_ values of 0.011 and 0.253 μl ml^−1^, respectively.

**Conclusion:**

Our results demonstrate that ROEO could have a potential role in the treatment of diseases related to infection by microorganisms or proliferation of cancer cells.

## Background

Various herb species have been used worldwide in prevention and treatment of diseases for many centuries. Today, several drugs have been developed from plants that are active against many common diseases [1]. Among the aromatic plants belonging to the *Lamiaceae* family, *Rosmarinus officinalis*, commonly known as Rosemary, is a flavoured herb which is native to the Mediterranean region and grows in various countries in the world [2]. The ROEO is a mixture of several major and minor compounds [3]. Debersac et al. [4] showed that 1,8-cineole is the major component of Rosemary essential oil, whereas, in other studies α-pinene was the main component, followed by 1,8-cineole, camphene, β-myrcene, borneol and camphor [5]. Essential oils represent an alternative “green “to be used in the cosmetic, pharmaceutical, agricultural and food fields while substituting chemical treatments. Biological activities of essential oils from various plants, including Rosemary, have been attributed to the presence of specific chemical compounds [6]. Several studies have demonstrated the antimicrobial [7], antiproliferative [8], anti-inflammatory [9] and antioxidant [10] activities of the ROEO. The oil of Rosemary is used as a biological preservative of food products [11] due to its antioxidant and antimicrobial properties [12].

Bacteria, during development, can be present in planktonic or sessile forms known as biofilm [13]. Biofilm is a microbial structure that causes many problems in medical field, in food industry and even to the environment. Thus, biofilm could cause many public health problems. Indeed, nearly 80% of chronic bacterial infections are associated with the presence of biofilm [14]. In addition, biofilms contaminate the majority of medical or prosthetic devices such as vascular prosthesis [15], urinary catheters [16], joint prostheses, pacemakers [17], contact lens [18] and dental plates [19]. As the antibiotic treatment is most effective against microbial infections, bacterial biofilms are highly resistant to antibiotics posing a public health problem. Indeed, concentrations of antibiotics needed to inhibit bacteria in biofilms may be greater than a thousand times that required to inhibit the same planktonic bacteria [20]. So, it is necessary to find alternatives to fight against biofilm problems. In addition, biofilms cause a problem in many food industries. They contaminate the surfaces in contact with food while causing an alteration of the organoleptic quality of food products [21]. It has been previously demonstrated that essential oils are able to penetrate and cause the destruction of microbial biofilms [22]. Few studies have shown the anti-biofilm activity of ROEO [23, 24]. However, all of these studies did not use *Staphylococcus epidermidis*, which is the major cause of catheter infections, as a model for anti-biofilm studies.

In many studies, antitumor activity has been reported for essential oils against several tumor cell lines. In fact, in-vitro anticancer activity of the essential oil isolated from the leaf of *Machilus mushaensis* from Taiwan was analyzed and the oil exhibited cytotoxic activity against human oral, liver, lung, colon, melanoma, and leukemic cancer cells [25]. Indeed, other researchers evaluated the anticancer activity of the essential oil of *Plectranthus amboinicus* (Lour) and this investigation exhibited the potent chemotherapeutic/chemopreventive effect of *P. amboinicus* (Lour) essential oil over lung metastasis [26].

In other studies, the oils of *Eucalyptus torquata* stems exhibited cytotoxic activities on MCF-7 cells [27]. Huang et al. [28] have investigated the effect of two volatile oils prepared by steam distillation (SD) and supercritical fluid extraction (SFE) of *Centipeda minima*, a Chinese herb used in the treatment of various diseases including cancer. They found that SFE oil displayed potent anti-angiogenic activity in a zebrafish model [28].

Taking all together, valorization of essential oils by the study of their original biological activities is of great interest. In this context, the aim of this study was to characterize the chemical composition of Tunisian ROEO, to evaluate its antibacterial, anti-biofilm activities and anticancer properties.

## Methods

### Essential oil

The plant material was collected from a house garden located at the region of Sfax, Tunisia. The ROEO was obtained after hydrodistillation using Clevenger-type apparatus operating at atmospheric pressure. The extracted oil was collected after decantation and dried over anhydrous sodium sulfate in the dark, then stored in sealed glass vials in a refrigerator at 4 °C prior to analysis and use.

### Cell lines and cultures

Human breast adenocarcinoma cell lines MCF-7 and Hela cell lines derived from cervical cancer were grown in DMEM supplemented with 10% fetal bovine serum, 50 IU ml^−1^ penicillin, 50 mg ml^−1^ streptomycin at 37 °C in a humidified 5% CO_2_ atmosphere.

### Gas chromatography/mass spectrometry (GC/MS) analysis

Analysis of the ROEO was performed using an Agilent 5975B mass spectrometer coupled to an Agilent 6890 N gas chromatograph using the protocol previously described by Boukhris et al. [29]. The identification of the ROEO compounds was realized using a comparison of their retention times to *n*-alkanes, and their similarities to MS corresponding database (Wiley and NIST Library) and published data and spectra of authentic compounds.

### Antibacterial activity

#### Determination of minimum inhibitory concentration (MIC)

The antibacterial activities of ROEO were investigated using micro-dilution method in 96-well plates as suggested by the National Committee for Clinical Laboratory Standards [30]. The test was carried out with two bacterial species *Staphylococcus aureus* ATCC 9144 and *Staphylococcus epidermidis* S61. Each bacterium was grown in LB medium overnight at 30 °C. The culture was then adjusted to an optical density of 0.6 at a wavelength of 600 nm. The essential oil was dissolved in dimethylsulfoxide (DMSO) to adequate concentrations and then filtered. A volume of 100 μl was added in each well of two columns of the plate with medium and successive two-fold dilution was prepared in plate to obtain final essential oil concentration of 10, 5, 2.5, 1.25, 0.61, 0.31, 0.15, 0.07 μl ml^−1^. An aliquot (20 μl) of the bacterial dilution was added to each well and a final volume of 200 μl/well was adjusted with medium. Wells containing only LB medium with inoculum and these containing medium, inoculum and Ampicillin served as controls. Microplates were incubated at 30 °C for 18–24 h. After incubation, bacterial growth was evaluated by the MTT revelation. 20 μl of 3- (4, 5-dimethyl-thiazol-2-yl)- 2,5-diphenyl-tetrazolium bromide (MTT) at concentration of 1 mg ml^−1^ was prepared in water, filtered and added to each well. The plates were incubated for 20 min in the dark with stirring. The viable bacteria were detected by the change of yellow MTT color to purple. The MIC was defined as the lowest EO concentration that completely inhibited any visual growth of the microorganisms being tested. So, the MIC interval was taken from the first well devoid of bacterial growth and the first well showing growth.

#### Determination of minimum bactericidal concentration (MBC)

To determine the MBC values, a volume of 10 μl from each well, which didn’t show an apparent growth as confirmed by MIC determination, was taken and plated on LB agar medium on the form of streaks. The plates were incubated at 30 °C for 24 h. The MBC was defined as the lowest essential oil concentration able to reduce and kill more than 99.9% of the initial inoculum.

### Determination of the effect of rosemary essential oil on *Staphylococcus epidermidis* S61 biofilm formation

#### Inhibition of initial cell attachment

Anti-biofilm activity was assessed in 96-well flat bottom plates using a method adapted by Nostro et al. [31] with some modifications. *Staphylococcus epidermidis* S61, a biofilm-forming strain in our Lab collection [32], was grown overnight in TSB (Tryptic Soy Broth) medium at 30 °C and diluted with fresh medium supplemented with 2.25% glucose. An aliquot of 100 μl of culture dilution was dispensed into each well to obtain a final OD_600 nm_ of 0.1. After, 100 μl of ROEO dissolved in TSB, containing 0.5% (v/v) of Tween 20, at different concentrations, were added into wells to reach final concentrations of 1.25, 2.5, 12.5 and 25 μl ml^−1^. wells containing TSB medium supplemented with 0.5% (v/v) of Tween 20 and inoculum without essential oil were served as controls. Plates were incubated for 24 h at 30 °C. After incubation, wells were emptied by tapping the plates into a disposal vessel. Planktonic cells were gently removed by washing each well twice with 200 μl of sterile phosphate buffered saline (PBS, pH 7.2). After washing, plates were dried at 60 °C for 1 h. Each well was stained with 150 μl of crystal violet solution prepared in 20% ethanol (v/v) for 15 min at room temperature. After incubation, the excess of crystal violet was eliminated and wells were rinsed three times with sterile water. Finally, 200 μl of glacial acetic acid (33% (v/v)) were added to each well and plates were incubated for 1 h at room temperature. All tests were performed in triplicate. Finally, the optical density (OD) of each well was measured using the microplate reader at a wavelength of 570 nm. In order to determine the ability of ROEO to prevent bacterial adherence and biofilm formation, the percentage of adherence inhibition was calculated using the following formula:

$$ {\left[\left(\mathrm{OD}\ \left(\mathrm{growth}\  \mathrm{control}\right)\hbox{--} \mathrm{OD}\ \left(\mathrm{sample}\right)\right)/\mathrm{OD}\ \left(\mathrm{growth}\  \mathrm{control}\right)\right]}^{\ast }100 $$

#### Effect on established biofilm

The effect of ROEO on established biofilm was studied using the method previously described by Kavanaugh and Ribbeck [33] with slight modification. After biofilm formation for 48 h, the medium and non-attached bacteria were removed by aspiration using a micropipette followed by a twice washing with PBS. 200 μl of ROEO, diluted in medium and supplemented with 0.5% (v/v) Tween 20, were placed into each well to yield final concentrations of 1.25, 12.5, 25 and 50 μl ml^−1^. Plates were further incubated at 30 °C for 24 h. After incubation, biofilms were stained with crystal violet as described previously. Each experiment was evaluated in triplicate. The control was a biofilm without essential oil. By comparing the OD values (570 nm) of the growth control with that of the essential oil, we calculated the percentage of biofilm eradication using the equation:

$$ {\left[\left(\mathrm{OD}\ \left(\mathrm{growth}\  \mathrm{control}\right)\hbox{--} \mathrm{OD}\ \left(\mathrm{sample}\right)\right)/\mathrm{OD}\ \left(\mathrm{growth}\  \mathrm{control}\right)\right]}^{\ast }100 $$

#### Viability quantification

The bacterial viability within the biofilm was quantified using the resazurin test. Resazurin is a blue fluorescent dye that is reduced by viable bacteria to fluorescent pink resorufin [34]. After biofilm formation for 48 h at 30 °C, planktonic bacteria were removed and the wells were washed twice with PBS. 200 μl of ROEO diluted in TSB medium with 0.5% Tween 20 (v/v), at different concentrations 1.25, 12.5, 25 and 50 μl ml^−1^ were added. TSB medium with 0.5% Tween 20 was used as control. After incubation during 24 h at 30 °C, TSB medium and non-adherent bacteria were removed followed by double washing with PBS.

For staining step, a volume of 100 μl of resazurin (7-hydroxy-3H-phenoxazine-3-one-10-oxide) (AppliChem GmBH, Germany) at a concentration of 10 μg ml^−1^ was added to each well. The plate was incubated in darkness and room temperature for 30 min. The fluorescence of the resorufin was then measured at a wavelength of 590 nm with an excitation at 550 of nm using a microplate reader (Varioscan Flash, Thermo Scientific).

### Fluorescence microscopy

Biofilm visualization was performed using fluorescence microscopy. Biofilms were grown on glass coverslips for 48 h. After, the medium was discarded and coverslips were washed with physiologic water to remove the non-adherent bacteria. Then, TSB medium containing 0.5% (v/v) Tween 20 was added in control wells and TSB (with 0.5% Tween 20) added with adequate volume of ROEO to assess the anti-biofilm activity of the oil. After 24 h, wells contained coverslips were emptied and washed twice with physiologic water. Acridine orange (0.1%, w/v, dissolved in PBS 1X) was used to stain biofilms formed in coverslips. Biofilms were observed under an OLYMPUS fluorescent microscope BX50 equipped with a digital camera OLYMPUS DP70 using U-MWB2 filter with excitation at 460–490 nm and emission at 520 nm.

### Anticancer activity

Cell viability of MCF-7 (human breast adenocarcinoma) and Hela (human cervical carcinoma) cell lines was assessed using the MTT assay as previously described by Mosmann [35]. This assay is based on the reduction of MTT into purple formazan crystals by the succinate dehydrogenase enzyme in the mitochondrial respiratory chain. Cells were harvested by trypsinization, seeded in 96 well-plates at 80000 cells per ml of medium and allowed to attach for 24 h. After, 100 μl of ROEO, first dissolved in DMSO and then in DMEM medium, were added to obtain final concentrations ranging from 0.004 to 1.1 μl ml^−1^ in wells. Control cells were supplemented with 100 μl medium with DMSO. MCF-7 cells were exposed to the treatment for 48 h. After treatment, the medium was removed and replaced with 100 μl of fresh medium supplemented with 10 μl of MTT solution (5 mg ml^−1^ in PBS). After 4 h, 100 μl of 10% SDS solution were added to each well. Subsequent to formazan dissolution, the optical density was evaluated at 570 nm using a multidetection microplate reader. The growth inhibition was expressed as follows:

(%) cell survival = (A1/A0)*100; where A0 is the control absorbance and A1 the absorbance of the treated cells.

### Cell migration assay

The cell migration assay was performed based on the protocol described by Dahham et al. [36] with slight modification. Briefly, MCF-7 and Hela cells were grown in DMEM medium supplemented with 10% (v/v) FBS until 80% of confluence at 37 °C and 5% CO_2_ in 60 mm tissue culture dish. Thereafter, two parallel wounds per dish were performed with a sterilized plastic tip and cells were washed twice with PBS 1X to remove cells in suspension. Upon adding medium, the treatment with adequate concentration of ROEO was added and incubated with an untreated sample as a control. Pictures of fixed positions in the wounds were taken with a Canon digital camera that was mounted on the inverted microscope (Leica DM IL) to follow the repopulation of the wound.

### Statistical analysis

All experiments were done in triplicate. The obtained results are expressed as mean values with the standard error. The statistical analyses were performed using Student’s t-test to compare the controls and treated samples at a significance level of 5%.

## Results

### Chemical constituents of ROEO

The composition of the ROEO is presented in Table 1 (percentage of each compound, Retention Time (RT) and Retention Index (RI)). Thirty-six compounds, corresponding to 99.9% of the total oil, were identified in the ROEO, according to their elution order on a DB-5MS column. ROEO contained a complex mixture of compounds. In fact, monoterpene hydrocarbons, with 62.28%, were the most important classes of compounds present in ROEO (Table 2). Other classes, oxygenated monoterpenes and sesquiterpene hydrocarbons, were also found in smaller amounts, 21.72% and 12.18%, respectively (Table 2). The remaining compounds were identified in traces. The major components of ROEO were 1,8-cineole (23.56%), camphene (12.78%), camphor (12.55%), β pinene (12.3%), γ-terpinene (7.56%) and caryophyllene oxide (5.02%) (Table 1).

**Table 1:** Chemical composition of ROEO

Peak number	Compound^a^	RT^b^	RI^c^	Area percentage (%)*
1	γ-Terpinene	6.74	1038	7.56
2	α-Terpinolene	7.07	1047	2.46
3	Camphene	7.28	1052	12.78
4	β-pinene	7.98	1070	12.30
5	1,8-cineole	10.13	1134	23.56
6	Sabinene	10.68	1153	0.49
7	Allyltoluene	11.15	1170	0.30
8	α-Pinene	11.41	1179	1.20
9	Linalool-L	12.02	1201	3.21
10	Camphor	12.83	1240	12.55
11	Pinocarvone	13.38	1267	0.84
12	Borneol	13.55	1275	2.53
13	Terpinene-4-ol	13.86	1290	1.23
14	Cryptone	14.05	1299	0.30
15	Myrtenol	14.43	1310	1.14
16	Verbenone	14.70	1317	0.81
17	Carveol	14.89	1323	0.19
18	Carvone	15.47	1339	0.48
19	Bornylacetate	16.63	1371	3.28
20	α-Cubebene	18.13	1415	0.22
21	α-Ylangene	18.70	1434	0.27
22	α-Copaene	18.85	1439	0.87
23	Sobrerol	19.23	1452	0.27
24	Eugenolmethylether	19.61	1464	016
25	Caryophyllene	20.05	1479	2.80
26	Germacrene-D	20.18	1483	0.19
27	Aromadendrene	20.43	1492	0.27
28	α-Caryophyllene	20.80	1505	0.67
29	α-Amorphene	21.35	1525	0.74
30	β-Selinene	21.57	1534	0.08
31	γ-Cadinene	21.76	1541	0.15
32	α-Muurolene	21.89	1546	0.17
33	β-Bisabolene	22.09	1553	0.14
34	γ-Cadinene	22.25	1559	0.34
35	Calamenene	22.44	1566	0.17
36	Caryophylleneoxide	24.12	1630	5.02
				99.9

^a^Compounds listed in order of their elution from a DB-5MS column

^b^Retention Time (minutes)

^c^Retention Index calculated against C8-C26 n-alkanes for DB-5MS column

*only the two first decimal places are presented

**Table 2:** Compound groups of ROEO

Compounds	%
Identified compounds	99.9
Monoterpene hydrocarbons	62.28
Oxygenated monoterpenes	21.72
Sesquiterpene hydrocarbons	12.18
Others	3.72

### Antibacterial activity

The antibacterial activity of the ROEO was evaluated by the micro-dilution method against two Gram positive bacterial strains, *Staphylococcus aureus* and *Staphylococcus epidermidis* which represent species that are commonly found in multi-resistant infections. ROEO produced inhibitory and bactericidal effects against the tested strains. Minimum Inhibitory Concentration (MIC) and Minimum Bactericidal Concentration (MBC) values are summarized in Table 3. ROEO exhibited important antibacterial effect against the two bacteria. It inhibited *Staphylococcus aureus* ATCC 9144 and *Staphylococcus epidermidis* S61 with MIC values in the range of [1.25–2.5] and [0.312–0.625] μl ml^−1^, respectively, whereas bactericidal activities against *Staphylococcus aureus* and *Staphylococcus epidermidis* reached higher concentrations values in the order of 5 and 2.5 μl ml^−1^, respectively.

**Table 3:** Minimum Inhibitory Concentration (MIC) and Minimum Bactericidal Concentration (MBC) of ROEO against *Staphylococcus* strains

	Antimicrobial activity	
Strains	MIC (μl ml^−1^)	MBC (μl ml^−1^)
*Staphylococcus aureus* ATCC 9144	[1.25–2.5]	5
*Staphylococcus epidermidis* S61	[0.312–0.625]	2.5

### Anti-biofilm activity

In this context, we investigated the ability of ROEO to inhibit biofilm formation or to eradicate preformed biofilm of *Staphylococcus epidermidis.*

**Fig. 1:**
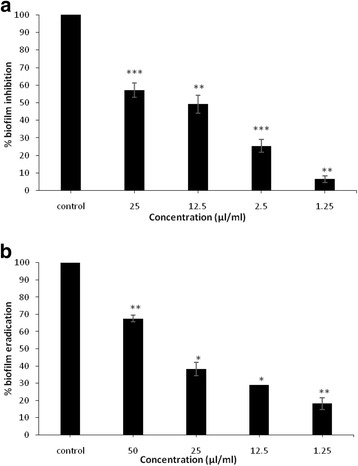
Effects of Rosemary essential oil on the inhibition of biofilm formation of *Staphylococcus epidermidis* (prevention) expressed as biofilm inhibition (%) (**a**) and on the reduction of established biofilm of *Staphylococcus epidermidis* expressed as biofilm eradication (%) (**b**). The results are presented as means ± SD of three independent experiments. .* *p* < 0.05, ** *p* < 0.01 or ***p < 0.001 versus control values

For the anti-adhesion activity, results showed an important attenuated level of *Staphylococcus epidermidis* biofilm formation with different concentration of ROEO ranging from 1.25 to 25 μl ml^−1^, which presented 4 to 80-fold greater than the MIC. At a concentration of 25 μl ml^−1^ (40–80 × MIC, 10 × MBC), ROEO showed the highest inhibitory effect with a percentage of inhibition value of 57.1%. Moreover, the doses of 12.5, 2.5 and 1.25 μl ml^−1^ exerted also an inhibitory effect with reduction rate of 49.21, 25.43 and 23.24%, respectively (Fig. 1a).

The ability of the essential oil to destroy established preformed biofilm was also investigated. After an overnight of incubation of established biofilm with essential oil, a concentration of 50 μl ml^−1^, which presented 20-fold MBC and between 80 and 160 × MIC, was able to reduce the preformed biofilm at only 67.53% (Fig. 1b). At a concentration of 12.5 μl ml^−1^, ROEO was able to inhibit 49.21% of cell attachment but only 29.05% of established biofilm was removed. So, the effect of essential oil against the preformed biofilm was lower than that of the inhibition of cell attachment.

**Fig. 2:**
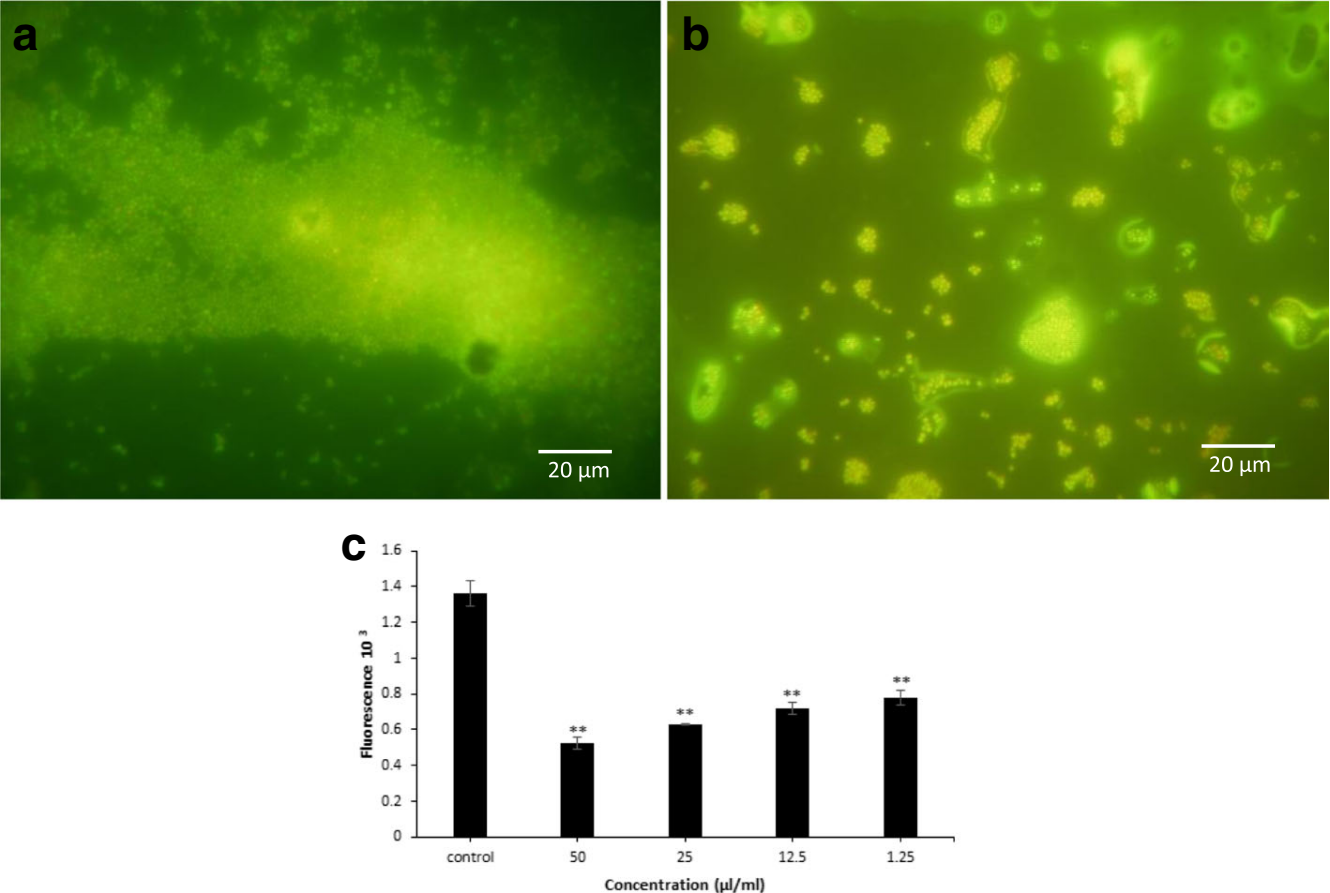
Fluorescence microscopy with biofilm coverslips. Biofilms were stained with acridine orange. Bar equals 20 μm **a** S61 biofilm, control coverslip; **b** S61 biofilm treated with 50 μl/ml of ROEO. **c** Viability assessment within *Staphylococcus epidermidis* biofilm challenged with various ROEO concentrations using resazurin assay. The results are presented as means ± SD of three independent experiments. ** *p* < 0.01 or ***p < 0.001 versus control values

Figure 2 shows two typical dimensional pictures obtained from the biofilm of strain S61 and the treated biofilm with ROEO using fluorescent microscopy. It could be concluded that ROEO exerted an anti-biofilm activity against S61 with an almost total destruction of biofilm matrix.

Moreover, Resazurin test showed that ROEO has an effect on the viability of *Staphylococcus epidermidis*. The cell viability is inversely proportional to the concentration of ROEO. In fact, at 50 μl ml^−1^, the cell viability in *Staphylococcus epidermidis* biofilm did not exceed 50% as assessed by fluorescence measurement (Fig. 2). The inhibition of cell viability was also found even at a concentration of 1.25 μl ml^−1^.

### Anticancer activity

**Fig. 3:**
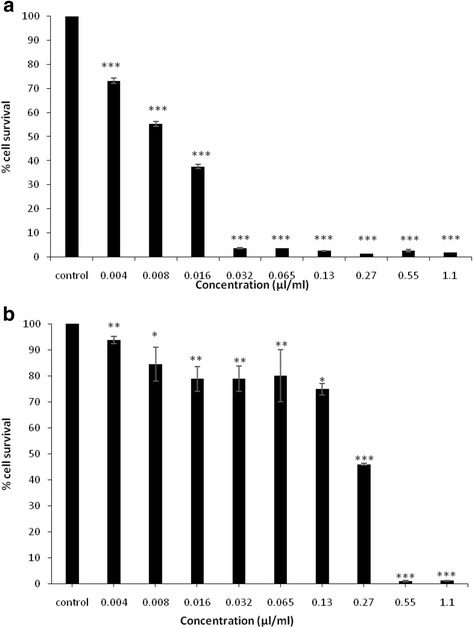
Effects of Rosemary essential oil on cancer cell lines. **a** Hela cells proliferation. **b** MCF-7 cells proliferation. The results are presented as means ± SD of three independent experiments. .* *p* < 0.05 or ***p < 0.001 versus control values

The ROEO was evaluated for its in vitro anticancer activities against human cancer cells using MTT assay. Both cell lines, Hela and MCF-7, were exposed to increasing concentrations of ROEO. Figure 3 shows the percentage of cell survival versus increasing concentrations of the essential oil in comparison with the control, which presents the untreated cells. As indicated in Fig. 3, the percentage of cell viability decreases with increasing essential oil concentrations. The IC_50_ values were calculated from the graphs. ROEO exhibited varying cytotoxic effect on Hela and MCF-7 cell lines with more pronounced activity against Hela cells. ROEO was strongly inhibiting the proliferation of Hela cells with an IC_50_ value of 0.011 μl ml^−1^, while at a concentration of 0.253 μl ml^−1^, it inhibited 50% of the MCF-7 cell proliferation.

### Analysis of wound healing and cell migration

**Fig. 4:**
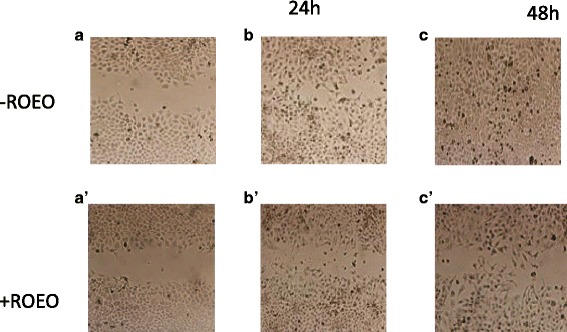
Analysis of cell migration by inducing artificial wound in Hela cells. **a**, **a**’: pictures of the wound in untreated and treated cells respectively at the beginning of the experience; **b**, **b**′: pictures of the wound in untreated and treated cells respectively after 24 h of incubation, **c**, **c**′: pictures of the wound in untreated and treated cells respectively after 48 h of incubation

In order to study cell migration and proliferation of untreated and treated cells with ROEO, the cell migration assay was performed. Results showed that the wound disappeared rapidly after 48 h for the untreated cells in comparison with the wound in the treated cells whose is slowly covered by the cells during the same incubation time for both Hela and MCF-7 cells (Fig. 4) (data not shown for MCF-7 cells).

## Discussion

In this research, we determined the chemical composition of the studied ROEO, its antibacterial, antibiofilm and cytotoxic activities. Various studies have been interested in the chemical composition of the ROEO from different countries. The results of ROEO composition are approximately in accordance with those found by Miladi et al. [37]. They reported that the basic compounds were 1,8-cineole (24.10%), camphor (19.87%) and camphene (8.65%), with a remarkably difference in the percentage of α- pinene (13.49%) and β- pinene (3.34%). In another report on ROEO from Brazil, 1,8-cineole (28.5%), followed by camphor (27.7%), α-pinene (21.3%) and camphene (8.7%) were reported as the predominant compounds [38]. Kadri et al. [39] studied the chemical composition of ROEO collected from the mount of Gafsa and reported the main one as 1,8-cineole (35.36%). However, it is different to the studied ROEO composition by the presence of important quantities of trans-caryophyllene (14.7%) and Borneol (9.97%). The quantitative variation of the ROEO chemical composition depends on several factors such as ecological conditions [40], geographic position [5], period of collection [41] and oil extraction method [42].

ROEO is rich in monoterpene compounds (62.28%), which were reported in the literature for their antimicrobial activities [43]. More particularly, the richness of the oil in monoterpene hydrocarbons such as 1,8-cineole (23.56%), characterized by its strong antimicrobial activity against several bacteria [44] may explain the inhibitory and bactericidal effect against both strains *S. epidermidis* and *S. aureus.* On the other hand, the effect of minor compounds such as Germacrene-D (0.19%), caryophyllene (2.8%), caryophyllene oxide (5.02%), terpinene-4-ol (1.23%), also known for their antimicrobial effects [44], could not be denied. In fact, it has been reported that these compounds could act synergistically and consequently be responsible of the founded antibacterial activity [45].

We evaluated the antibiofilm activity against *Staphylococcus epidermidis*, which is affiliated to coagulase-negative staphylococci and known as a commensal microorganism on the human skin [46]. According to the CDC National Nosocomial Infections Surveillance (NNIS) System Report [47], *Staphylococcus epidermidis* is the most common cause of nosocomial infections in patients with all types of medical devices. In fact, it is often isolated from infected prosthetic joints, central venous catheters, intracardiac devices, artificial heart valves and vascular grafts [48]. Chu et al. [49] demonstrated that *S. epidermidis* is the cause of 13% of prosthetic valve endocarditis (PVE) infections, with a high rate of intracardiac abscess (38%) and mortality by 24%. Billions of dollars are spent each year for the replacement of infected devices such as intravascular and urinary catheters, mechanical heart valves, pacemakers, contact lenses, prosthetic implants [50].

Two attachment stages are required for biofilm formation; the first is the reversible attachment (low) followed by the second which is the irreversible attachment (strong). Therefore, the high required concentrations to eradicate the already established biofilm compared to those needed to inhibit biofilm formation can deduce that the preformed biofilm may be in the irreversible attachment phase [51]. On the other hand, this resistance can be attributed to the three-dimensional architecture of the mature biofilm and the presence of an exopolysaccharide polymer matrix acting as a physical barrier against the penetration of antimicrobial agents [52].

The comparison of the effect of ROEO against biofilm and planktonic cells showed that bacterial cells within biofilm were more resistant than their planktonic forms. These findings correlate with previous studies showing that bacterial resistance to antimicrobial agents within the biofilm can reach 1000 times of the bacterial resistance in suspension [20]. Another factor, which can justify the biofilm resistance, is that the majority of antimicrobial agents are active against cells in active division, which is not the case for bacteria in biofilm mode [53]. In fact, the biofilm is a stack of layers. The deepest are characterized by low growth rates due to the lack of oxygen and nutrients [52, 54]. It has been previously reported that essential oils could diffuse through polysaccharide matrix of the mature biofilm and destabilize it due to strong intrinsic antimicrobial activities [31]. Moreover, the anti-adherent activity is explained by the alteration of bacterial surface proteins due to their interactions with oils. This, will inhibit the initial attachment phase to the abiotic surface [31]. These results support the medical application of these oils for the prevention and/or treatment of certain infections and diseases.

ROEO strongly inhibited the proliferation of Hela followed by MCF-7 cells. The variability of cytotoxicity effect of this oil against Hela and MCF-7 cells could be associated to the polyvalent oil composition, the different types of interaction between compounds and cells [55] and the difference of cancer cells sensitivities to cytotoxic compounds [56]. According to the literature, the anticancer activity of ROEO is due to the main components such as 1,8-cineole, camphor, α-pinene [57, 58] and β-caryophyllene [59]. The evaluation of cell migration inhibition by ROEO showed that the oil could prevent the rapid cell migration. We can conclude therefore that the ROEO slows the proliferation and the cell migration. Thus, ROEO could be considered as a potential anti-cancer oil.

## Conclusions

The present study has reported composition and biological activities of ROEO including anti-biofilm and anti-cancer activities. The ROEO effectively controlled the growth and biofilm formation of Gram-positive *S. epidermidis*. In addition, the ROEO also showed anticancer activity against human cancer MCF-7 and Hela cells. The study concludes that ROEO could be used as potential therapeutics against human cancer and control of pathogenic bacteria.

## Abbreviations

DMSO: Dimethylsulfoxide
GC-MS: Gas chromatography/mass spectrometry
MBC: Minimum bactericidal concentration
MIC: Minimum inhibitory concentration
MTT: 3- (4, 5-dimethyl-thiazol-2-yl)- 2,5-diphenyl-tetrazolium bromide
ROEO: *Rosmarinus officinalis* essential oil
*S. aureus*: *Staphylococcus aureus*
*S. epidermidis*: *Staphylococcus epidermidis*
TSB: Tryptic Soy Broth
